# Chemical composition and biological activities of *Helicteres vegae* and *Heliopsis sinaloensis*

**DOI:** 10.1080/13880209.2017.1306712

**Published:** 2017-03-28

**Authors:** Sandra Olivas-Quintero, Gabriela López-Angulo, Julio Montes-Avila, Sylvia Páz Díaz-Camacho, Rito Vega-Aviña, José Ángel López-Valenzuela, Nancy Yareli Salazar-Salas, Francisco Delgado-Vargas

**Affiliations:** a School of Chemical and Biological Sciences, Autonomous University of Sinaloa, Culiacan, Sinaloa, Mexico;; b School of Agronomy, Autonomous University of Sinaloa, Culiacan, Sinaloa, Mexico

**Keywords:** Phenolics, antimicrobial, antimutagenic, antioxidant, flavonoids, liquid chromatography, electrospray ionization, mass spectrometry, toxicity

## Abstract

**Context:***Helicteres vegae* Cristóbal (Sterculiaceae) (Hv) and *Heliopsis sinaloensis* B.L. Turner (Asteraceae) (Hs) are endangered and poorly studied plant species; related plants have been used against chronic-degenerative and infectious diseases. Therefore, Hv and Hs could be sources of bioactive compounds against these illnesses.

**Objective:** To determine the chemical composition and biological activities (antioxidant, antimutagenic and antimicrobial) of Hv and Hs leaves (L) and stems (S).

**Materials and methods:** Methanol extracts (ME) of each plant/tissue were evaluated for their phytochemicals; phenolics (HPLC-DAD-ESI-MS); antioxidant activity (AA) (0.125–4 mg/mL) (DPPH, ABTS, ORAC and β-carotene discoloration); antimutagenicity (0.5 and 1 mg/plate) (Ames assay, tester strain *Salmonella enterica* serovar Typhimurium YG1024, 1-nitropyrene as mutagen); activity against human pathogens (1 mg/mL); and toxicity (0.01–2 mg/mL) (*Artemia salina* assay).

**Results:** All ME showed flavonoids and triterpenes/steroids. The ME-SHv had the highest content of total phenolics (TP) (2245.82 ± 21.45 mg GAE/100 g d.w.) and condensed tannins (603.71 ± 1.115 mg CE/100 g d.w.). The compounds identified were flavonoids (kaempferol 7-*O*-coumaroylhexoside, and two kaempferol 7-*O*-rhamnosylhexosides) and phenolics [rosmarinic acid, and 3′-*O*-(8″-*Z-*caffeoyl) rosmarinic acid]. The ME-LHs showed the highest content of flavonoids (357.88 mg RE/g d.w.) and phenolic acids (238.58 mg CAE/g d.w.) by HPLC. The ME-SHv showed the highest AA. All ME were strong antimutagens (63.3-85.7%). Only the Hs extracts were toxic (ME-LHs, LC_50_ = 94.9 ± 1.7 μg/mL; ME-SHs, LC_50_ = 89.03 ± 4.42 μg/mL).

**Discussion and conclusions:** Both Hv and Hs are potential sources of preventive and therapeutic agents against chronic-degenerative diseases.

## Introduction

Chronic-degenerative diseases (CDD) represent serious public health problems worldwide. However, the pharmacological therapies available for the treatment of these illnesses are limited and most of them are inefficient. Plants have been traditionally used for the treatment of several diseases, and therefore they represent an important source of bioactive compounds against CDD. Phenolics are among these metabolites; they are associated with many biological properties (e.g., antioxidant and antimutagenic) of plant extracts (Cervellati et al. 2002; Zhu et al. 2014), and characterized from complex plant mixtures by liquid chromatography coupled with mass spectrometry (HPLC-DAD-ESI-MS) (Ablajan et al. 2006).

Mexico is ranked fifth in the world in terms of floristic diversity, but many of these plants are unknown and only few of those catalogued have been scientifically studied. *Helicteres vegae* Cristóbal (Sterculiaceae) and *Heliopsis sinaloensis* B.L. Turner (Asteraceae) are endemic plants to the state of Sinaloa, which is located in northwestern Mexico. These plant species have not been previously studied; however, other members of these genera have been used to treat CDD and infectious diseases (Molina-Torres et al. 1999; Varghese et al. 2012), and specific compounds have been associated with such biological activities (Arriaga-Alba et al. 2013).

In this study, we carried out a chemical characterization and evaluated the antioxidant, antimutagenic and antimicrobial activities of methanol extracts (ME) of *Helicteres vegae* and *Heliopsis sinaloensis* to demonstrate their potential use as novel sources of pharmaceutical compounds or supplements for the treatment and prevention of chronic-degenerative and infectious diseases.

## Materials and methods

### Plant material

*Helicteres vegae* was collected during September 2011 from ‘El Saladito’, municipality of Elota, Sinaloa (80 masl, N 23°51′24″, W 106°47′42″), and *Heliopsis sinaloensis* was obtained during July 2011 from ‘Imala’, municipality of Culiacan, Sinaloa (100 masl, N 24°51′37″, W 107°13′01″). Plant collectors were Vega-Aviña R, Delgado-Vargas F and Pío-León JF. Plant specimens were identified by Vega-Aviña R and deposited in the herbarium of the Agronomy School, Autonomous University of Sinaloa, with the assigned numbers 11805 (*Helicteres vegae*) and 11816 (*Heliopsis sinaloensis*). Leaves and stems were recovered from each plant, freeze-dried (freeze dryer VirTis 25EL, VirTis Co., Gardiner, NY), and milled to get a flour that passed through a number 40 sieve. Flours were stored at −20 °C in darkness until use.

### Reagents and solvents

The reagents of analytical grade were purchased from Sigma/Aldrich (St. Louis, MO): β-carotene, 2,6-di-tert-butyl-4-methylphenol (BHT), 2,2-azino-bis(3-ethylbenzothiazoline-6-sulfonic acid) diammonium salt (ABTS), 2,2-diphenyl-1-picrylhydrazyl (DPPH), 6-hydroxy-2,5,7,8-tetramethylchromane-2-carboxylic acid (Trolox), Folin-Ciocalteu reagent, disodium fluorescein, 2,2-azobis(2-amidinopropane) dihydrochloride (AAPH), chemical standards (gallic acid, rutin hydrate, caffeic acid, kaempferol, and catechin), 1-nitropyrene (1-NP), dimethyl sulfoxide (DMSO). HPLC grade organic reagents were from Baker Inc. (Philipsburg, PA).

### Microorganisms

Tester strain YG1024 from *Salmonella enterica* serovar *typhimurium* was kindly provided by Dr. Takehiko Nohmi, Division of Genetics and Mutagenesis, Biological Safety Research Center, National Institute of Hygienic Science, Japan. The strain was maintained, propagated, routinely tested for genetic markers, and re-isolated whenever necessary. Eleven human pathogen bacterial strains were used for the antibacterial assay: four ATCC (*Staphylococcus aureus* 29213, *Enterococcus faecalis* 29212, *Escherichia coli* 25922, and *Pseudomonas aeruginosa* 27853) (DIFCO Laboratories, Detroit, MI); and seven clinical isolates (*Streptococcus aureus*, *Streptococcus* group A, *Salmonella enterica* serovar *typhi*, *Shigella dysenteriae*, *E. coli* A011, *E. coli* A019 and *E. coli* A055), which were provided by the Laboratory of Bacteriology of the National Institute of Pediatrics, Mexico City, Mexico. *Giardia lamblia* WB strain was provided by the Department of Experimental Pathology, Center for Research and Advanced Studies of the National Polytechnic Institute, Mexico City, Mexico. *Artemia salina* cysts were from a commercial trademark (Brine shrimp eggs, Bio-Marine Inc, Hawthorne, CA).

### Preparation of ME

Flours were extracted with methanol (1:10 w/v) for three days at 150 rpm/room temperature; supernatant was recovered by filtration daily, and the residue was extracted again with fresh solvent. Supernatants were mixed and the solvent was removed with a rotavapor (Büchi Labortechnick AG, Flawil, Switzerland) at 40 °C; the residues were freeze-dried to obtain the ME of leaves (L) and stems (S) of *Helicteres vegae* (Hv) and *Heliopsis sinaloensis* (Hs). The extraction yields (%) of the ME were: ME-LHv 14.12, ME-SHv 12.73, ME-LHs 25.02, and ME-SHs 13.50. Extracts were stored at −20 °C in darkness until use.

### Phytochemical analysis

Assays for phytochemicals in ME were carried out in test tubes or by thin-layer chromatography (silica gel matrix with fluorescent indicator 254 nm, Sigma/Aldrich) as follows: the Salkowski reaction for terpenes/sterols; the Shinoda test for flavonoids; reaction with 1.0% gelatin solution and quinine sulphate solution with FeCl_3_ for tannins; lather formation for saponins; yellow fluorescence by reaction with NaOH for coumarins; the Borträger reaction for free anthracenics; the reagents of Dragendorff and Mayer and Wagner for alkaloids; and the reagents of Baljet, Raymond-Marthoud, Keller-Killiani, Lieberman-Burchard, and Salkowski for cardiotonics (Harborne 1980; Yawalikar et al. 2014). The results were reported as the presence (+) or absence (−) of each family of compounds in the ME.

### Phenolics determined by colorimetric methods

Total phenolics (TP) were measured by the Folin-Ciocalteu method as described by Ahumada-Santos et al. (2013). A 0.02 mL aliquot of ME (1–2 mg/mL), 1.58 mL of distilled water, and 0.1 mL of 2 N Folin-Ciocalteu reagent were mixed in a test tube. The mixture was left stand at 40 °C for 5 min, then added with 0.3 mL of Na_2_CO_3_ saturated solution, mixed, incubated at 40 °C in darkness for 30 min, and the absorbance was measured at 765 nm. A calibration curve of gallic acid (0–500 μg/mL in methanol) was used to report the TP as milligrams of Gallic Acid Equivalents per 100 g of flour on a dry weight basis (mg GAE/100 g d.w.).

Condensed tannins were quantified as described by Bae et al. (1993). In a test tube (12 × 75 mm), 0.5 mL of ME (5–10 mg/mL) was vortex mixed with 3 mL of butanol:HCl (95:5 v/v) and 0.1 mL of ferric reagent (2% of ferric ammonium sulfate in 2 N HCl, 50:50 v/v). The mixture was heated at 97–100 °C for 60 min, whereas that used as blank was kept at room temperature. The mixture was brought to room temperature before measuring the absorbance at 550 nm. A calibration curve of catechin (0–5 mg/mL in methanol) was used to report the content of condensed tannins as milligrams of Catechin Equivalents per 100 g of flour d.w. (mg CE/100 g d.w.).

### Phenolic compounds determined by HPLC-DAD-ESI-MS

Quantitation was carried out by the internal standard method. The ME (20 mg) was mixed with 100 μL of caffeic acid (1 mg/mL), 350 μL of rutin (1 mg/mL), and 5 mL of deionized water. The mixture was sonicated for 50 min and the phenolic compounds were extracted three times with 5 mL of HPLC grade ethyl acetate. The solvent was removed with a rotavapor at 40 °C, whereas the residue was re-suspended in HPLC grade methanol (1:1 w/v), sonicated and passed through a syringe filter (PVDF membrane 0.45 μm, HPLC certified, Thermo Scientific, Darmstadt, Germany). A 5 μL aliquot was injected into the HPLC-DAD system (ACCELA, Thermo Scientific, Waltham, MA). The separation was carried out using a Fortis C18 HPLC column (3 μm, 50 × 2.1 mm) (Fortis Technologies Ltd, Neston, UK) with a linear gradient of 1% (v/v) formic acid (A) and acetonitrile (B), 0.5–60% of B in 35 min, and a total running time of 50 min.

The HPLC-DAD was coupled to a mass spectrometer with an electrospray ionization source (ESI) (Thermo Scientific, LTQ XL, US). The analysis was carried out in negative mode and full scan spectra were obtained in the *m/z* range of 50–1500. The parameters of the capillary tube were 35 V and 300 °C. Nitrogen and helium gases were used for drying and collision, respectively. The results were analyzed with the Xcalibur 2.2 software (Thermo Scientific, US).

Direct sample insertion was used for the MS^n^ experiments, scanning was in negative mode and the selected ion was fragmented by collision induced dissociation applying 10–45 V.

The identification of phenolics was based on the UV-spectra, MS fragmentation, and by comparisons with MS data both generated with commercial standards and reported in the literature. Flavonoids were quantified as Rutin Equivalents (mg RE/g d.w.) and phenolics as Caffeic Acid Equivalents (mg CAE/g d.w.).

### Antioxidant activity

#### DPPH method

Antioxidant activity by DPPH was measured according to Brand-Williams et al. (1995) with minor modifications. Aliquots of 0.2 mL of ME (0.25–4 mg/mL) or Trolox (0–75 μg/mL) and 1.8 mL of 150 μM DPPH were mixed in test tubes. The mixture was homogenized, left to stand at 37 °C/darkness for 30 min and the absorbance was measured at 515 nm. The results were reported as Trolox Equivalents Antioxidant Capacity (TEAC) per gram of flour on a dry weight basis.

#### ABTS method

The antioxidant activity by ABTS was measured according to Re et al. (1999). The radical (ABTS^•+^) was produced by mixing 5 mL of ABTS (14 mM in water) and 5 mL of potassium persulfate (4.9 mM); the mixture was left to stand at room temperature/darkness for 12–16 h. ABTS^•+^ was diluted with phosphate buffer saline (PBS) (pH 7.4, 23 mM) to reach an absorbance of 0.7 ± 0.02 at 734 nm. For evaluation, 0.05 mL of ME (0.125–4 mg/mL) or Trolox (0–0.4 mg/mL) was mixed with 1.95 mL of the ABTS^•+^ dilution; the mixture was left to stand at 37 °C for 10 min and the absorbance was measured at 734 nm. The results were expressed as TEAC per gram of flour on a dry weight basis.

#### ORAC method

Oxygen Radical Absorbance Capacity (ORAC) was measured as described by Huang et al. (2002). ME (2 mg/mL in methanol) was mixed with PBS (pH 7.4, 23 mM) to obtain 1:50, 1:100, 1:200 and 1:300 dilutions. Aliquots of 25 μL of the diluted ME or Trolox (0–100 μmol/L) were added to a 96 microwell plate and placed into a fluorescence spectrophotometer (Synergy HT, Bio-TEK Instruments, Winooski, VT). The equipment added 150 μL of fluorescein (0.1 μM in PBS), mixed the microplate at 1200 rpm for 20 s, and then added 25 μL of AAPH (0.207 g in 5 mL of PBS). The reaction was carried out at 37 °C and the fluorescence intensity (485 nm (ex)/538 nm (em)) was measured every minute up to 40 min. The area under the curve (AUC) was calculated as AUC =0.5 + *f*_1_/*f*_0_ +⋯+ *f_i_*/*f*_0_ +⋯+ *f*_39_/*f*_0_ + 0.5(*f*_40_/*f*_0_*)*, where *f*_0_ is the fluorescence at 0 min and *f_i_* that measured at time *i* (*i* = 1, 2, 3, … 40 min). Results were expressed as TEAC per gram of flour on a dry weight basis.

#### β-Carotene discoloration

The β-carotene discoloration (βCD) method reported by Wang et al. (2006) was used with minor modifications. Briefly, the reaction mixture was prepared by homogenization of 50 mg of Tween 40, 6.25 μL of linoleic acid and 500 μL of β-carotene (2 mg/mL in CH_2_Cl_2_); the solvent was eliminated with a stream of N_2_(g) and the residue was vortex-mixed with 25 mL of 30% H_2_O_2_. A mixture without β-carotene was used as blank. The assays were carried out in 96 well flat bottom plates. For the blank, 50 μL of DMSO were mixed with 250 μL of the blank mixture; to evaluate the extracts, 50 μL aliquots of the ME or butylhydroxytoluene (BHT) (0.5 and 1 mg/mL in DMSO) or DMSO were mixed with 250 μL of the reaction mixture. Absorbances were determined at 490 nm with a microplate reader (Multiskan Bichromatic, Thermo Fisher Scientific, USA) at 0 min and after 2 h of incubation at 50 °C. The rate of β-carotene degradation (*R*) was determined as *R* = (ln [*a*_S-C_/*b*_S-C_])/t; where, a_S-C_ is the absorbance of the sample (S) or control (C) at 0 min, and *b*_S-C_ is the absorbance of S or C at 120 min. Antioxidant activity was calculated as percentage of inhibition of β-carotene discoloration: % AA = [(*R*_control_ − *R*_sample_)/*R*_control_] × 100.

#### Antimutagenic activity

Antimutagenicity was evaluated by the Ames microsuspension assay, as described by Cano-Campos et al. (2011), using the tester strain *Salmonella enterica* serovar *typhimurium* YG1024 and 1-nitropyrene (1-NP) as mutagen. The toxicity and mutagenicity of the ME were evaluated up to 1 mg/tube.

*SalmonellaTyphimurium* was grown overnight in a metabolic bath 3540 (Lab-Line Instruments, Inc, Melrose Park, IL) at 37 °C to reach about 1–2 × 10^9^ cells/mL; the culture medium was a mixture of 50 mL of Oxoid nutritive broth number 2 (Oxoid Ltd, Hants, UK) with 157.5 μL of ampicillin (8 mg/mL). Cells were recovered by centrifugation at 4500 rpm/4 °C for 10 min and the pellet was re-suspended (1 × 10^10^ cells/mL) in PBS (0.15 M, pH 7.4). The culture medium used for the assay was prepared in a sterile test tube (12 × 75 mm) kept on ice, and the ingredients were added as follows: 0.095 mL of cocktail (38.5 μL of water, 50 μL of 0.2 M NAP buffer pH 7.4, 2 μL of a mixture of 0.4 M KCl and 1.65 M MgCl_2_, 4 μL of 0.1 M NADP^+^, and 0.5 μL of 1 M d-glucose-6-phosphate), 0.1 mL of bacteria (1 × 10^10^ cells/mL), 0.005 mL of 1-NP (100 ng/tube), and 0.01 mL of ME (0.5 and 1 mg/tube) or DMSO; 0.015 mL of DMSO was used for the negative control. The mixture was incubated at 150 rpm/37 °C for 90 min, then placed on ice, vortex-mixed with 2 mL of molten top agar supplemented with 90 nmol of biotin/histidine and poured into minimal glucose agar plates. Plates were incubated at 37 °C for 48 h and the colonies counted (SOL-BAT Model Q-20, SOL-BAT Co., Puebla, Mexico). The inhibition of the 1-NP mutagenicity was determined as % inhibition = (1–*A*/*B*) × 100; where *A* is the number of revertants in the presence of the extract and 1-NP and *B* is in the number of revertants when only 1-NP was present in the mixture.

Antimutagenic activity was classified based on the % inhibition as: 0–20% (negative), 20–40% (weak), 40–60% (positive or moderate), 60–90% (strong) and >90% (suspected toxicity) (Wall et al. 1988).

The toxicity or mutagenicity of ME was determined by using the mutagenic index (MI) at each concentration: MI=(Rs/Rws); where *Rs* and *Rws* are the average number of revertants/plate with and without the evaluated sample, respectively. The sample was considered mutagenic if MI ≥2 or cytotoxic if MI ≤0.6 (Maron & Ames 1983; Rosa et al. 2006).

#### Antimicrobial activity

Antibacterial activity was evaluated against Gram negative and Gram positive human pathogenic bacteria, four ATCC control and seven clinical isolates strains. Evaluation was carried by the microdilution method, as described by the Clinical and Laboratory Standards Institute (CLSI 2012), and using up to 1 mg/mL of ME. The assay was carried out in U-bottom 96 microwell plates, containing in each well 50 μL of inoculum (5 × 10^5^ UFC/mL) and 50 μL of the ME (DMSO 2% v/v Mueller Hinton). The positive control contained gentamicin (0.25–16 μg/mL) instead of the ME. The microwell plate was incubated (37 °C/18–24 h) and the Minimal Inhibitory Concentration (MIC) was determined by visual examination.

Antiparasitary activity against *Giardia lamblia* trophozoites was carried out as reported by Calzada et al. (1998). In a disposable tube, 1 mg of ME was resuspended in 900 μL of TYI-S 33 modified medium with DMSO (0.5% v/v) and then mixed with 100 μL of inoculum (1 × 10^6^ trophozoites/mL). Metronidazole (Sigma) was used as standard anti-giardiasis. After incubation for 24 h at 37 °C/5% CO_2_, cell viability was measured using a mitochondrial dehydrogenase activity assay with the substrate 3-(4,5-dimethylthiazol-2-yl)-2,5-diphenyltetrazolium bromide (MTT). Viable cells reduce MTT and the absorbance of the formed product was measured at 540 nm (Multiskan Bichromatic, Thermo Fisher Scientific, US).

#### Toxicity

*Artemia salina* eggs (100 mg) were suspended in 250 mL of NaCl solution (38 g/L) and incubated at 25–30 °C for 48 h. Nauplii were recovered and placed in fresh saline solution. ME dissolved in DMSO (40 mg/1400 μL of 10% DMSO) was used to prepare serial dilutions (10–2000 ppm/tube) in saline solution. The evaluation was carried out in test tubes. Each tube was filled with 10 nauplii, 140 μL of each ME dilution or DMSO (10% v/v) and saline solution up to 2 mL. Tubes were incubated at room temperature (25–30 °C) for 24 h and the number of living nauplii/tube was counted (Meyer et al. 1982; Solis et al. 1993). Probit analysis was used to determine the values of the lethal concentration 50 (LC_50_). Toxicity scale in the *A. salina* assay was registered as high (0.1–100 μg/mL), moderate (100–300 μg/mL), low (300–640 μg/mL), minimal (>640 μg/mL) or nontoxic (>2000 μg/mL) (Meyer et al. 1982; Sanabria-Galindo et al. 1997).

#### Statistical analysis

Data were analyzed by one-way analysis of variance and the differences among means were established by the Fisher test (LSD, *α* = 0.05). Pearson correlation analysis was used to determine the association between variables. Statistical analyses were performed using the software STATGRAPHICS Centurion XVI (Statpoint Inc., Warrenton, VA). All evaluations were done at least by triplicate.

## Results

### Chemical composition

#### Phytochemical analysis

Five of the nine families of the secondary metabolites analyzed were found in the ME (Table 1). ME-SHs and ME-LHv showed the highest and the lowest diversity of metabolites, respectively. Colorimetric assay suggested tannins are highly abundant in the ME-SHv.

**Table 1.** Secondary metabolites of methanol extracts (ME) from leaves (L) and stems (S) of *Helicteres vegae* (Hv) and *Heliopsis sinaloensis* (Hs)^a^.

Family of compounds	ME-LHv	ME-SHv	ME-LHs	ME-SHs
Alkaloids	–	–	–	–
Reducing sugars	+	–	+	+
Cardiotonics	–	–	–	–
Volatile coumarins	–	–	–	–
Free anthracenics	–	–	–	–
Flavonoids	+	+	+	+
Saponins	–	+	–	+
Tannins	–	+	+	+
Triterpenes or steroids	+	+	+	+

a The metabolites are present (+) or absent (−) in the corresponding methanol extract.

#### Phenolics determined by colorimetric methods

Significant differences in TP content (mg GAE/100 g d.w.) were found among the analyzed ME; ME-SHv showed the highest content (2245.82 ± 21.45) followed by ME-LHs (1604.65 ± 118.25), ME-SHs (580.70 ± 30.68) and ME-LHv (571.85 ± 42.64). The colorimetric determination of condensed tannins was only possible in the ME of *H. vegae* stems (ME-SHv) (603.71 ± 1.115 mg CE/100 g d.w.).

#### Phenolic compounds determined by HPLC-DAD-ESI-MS

**Figure 1.**
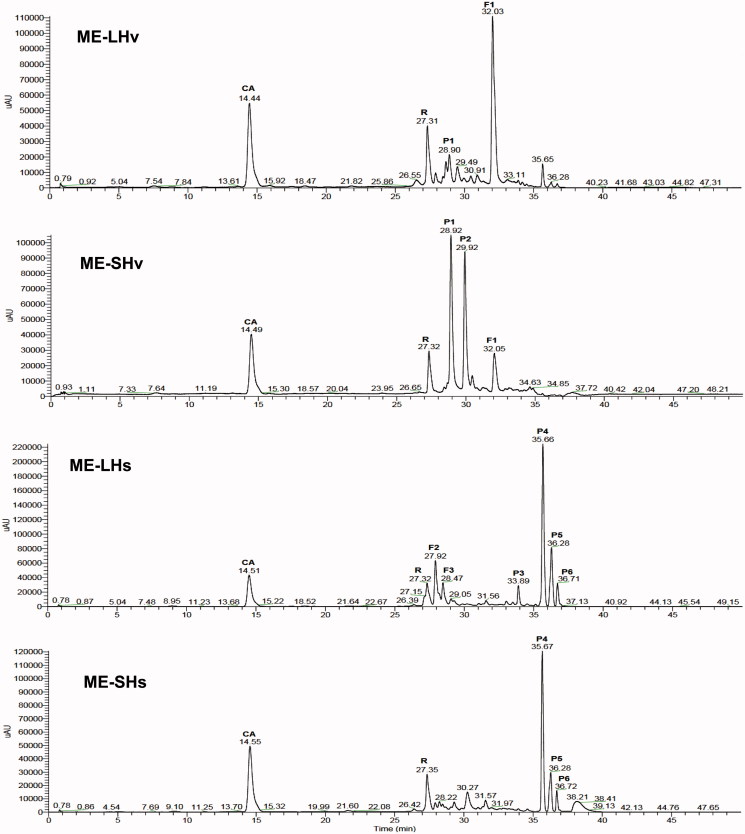
HPLC-DAD chromatograms of the methanol extracts (ME) from leaves (L) and stems (S) of *Helicteres vegae* (Hv) and *Heliopsis sinaloensis* (Hs). The identity of the major phenolics (flavonoids F and phenolic acids P) is shown in Table 1. Peaks for the commercial standards are CA (caffeic acid) and R (rutin).

HPLC analyses showed that flavonoids (F) and phenolic acids (P) were the major compounds of the ME (Figure 1). The main phenolics of *H. vegae* and *H. sinaloensis* were different, and some of them were tissue specific: P2 was only found in the ME-SHv, whereas F2, F3 and P3 were specific for the ME-LHs. In addition, several phenolics were found in both tissues of the same plant but at different concentrations: P1 and F1 in *H. vegae*, and P4, P5 and P6 in *H. sinaloensis* (Figure 1).

**Figure 2.**
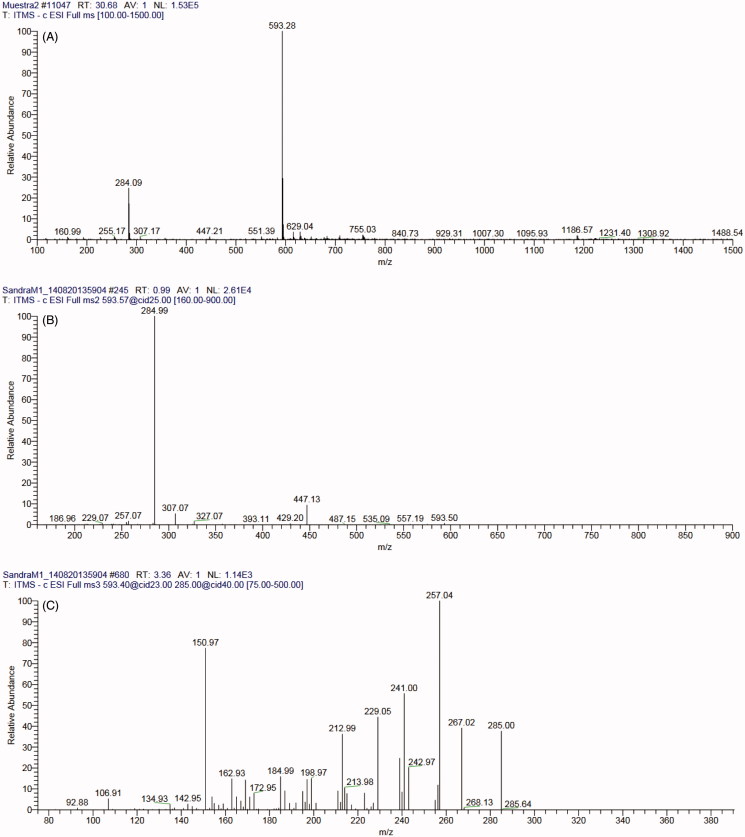
ESI-MS^n^ in negative mode of the compound F1. Full scan (A) and fragmentation of ion *m/z* 593, MS^2^ (B) and MS^3^ (C).

The analysis of the retention times, absorption spectra, ESI-MS fragmentation patterns (Table 2; Figures 2, 1S, 2S and 3S) and ESI-MS data generated with commercial standards (i.e., flavonol aglycones and caffeic acid) allowed the identification of five phenolics: three kaempferol derivatives (kaempferol 7-*O*-coumaroylhexoside and two kaempferol 7-*O*-rhamnosylhexosides) and two phenolic acids [rosmarinic acid and 3′-*O*-(8″-*Z-*caffeoyl) rosmarinic acid] (Table 2).

**Table 2.** HPLC-DAD-ESI-MS characterization of phenolics in methanol extracts (ME) from leaves (L) and stems (S) of *Helicteres vegae* (Hv) and *Heliopsis sinaloensis* (Hs)^a^.

Peak	*t*_R_ (min)	UV *λ*_máx_ (nm)	[M – H]^−^*m/z*	ESI-MS*^*n*^ m/z* (% relative abundance)	Tentative identification	Extract of plant/tissue
F1	32.05	316, 266, 242	593	MS^2^ [M – H]^-^: 593→447 (12.2), 307 (5), 285 (100) MS^3^ [M – H]^-^: 593→285→267 (30), 257 (100), 255 (4), 241 (50), 229 (45), 213 (43), 169 (20), 151 (83), 107 (7.5)	Kaempferol 7-*O-* coumaroylhexoside	ME-LHv, ME-SHv
F2	27.94	346, 265, 240	593	MS^2^ [M – H]^-^: 593→447 (2), 327 (6), 285 (100), 257(6) MS^3^ [M – H]^-^: 593→285→285 (98), 267 (50), 257 (100), 255 (3), 229 (43), 213 (28), 197 (18), 195 (10), 169 (5), 163 (18), 151 (15)	Kaempferol 7-*O-*rhamnosylhexoside	ME-LHs
F3	28.47	345, 265, 240	593	MS^2^ [M-H]^-^: 593→447 (2), 285 (100), 283 (3) MS^3^ [M-H]^-^: 593→285→257 (100), 255 (4), 229 (41), 213 (22), 195 (7), 162 (15.8), 151 (12)	Kaempferol 7-*O-*rhamnosylhexoside	ME-LHs
P1	28.93	328, 247, 232	359	MS^2^ [M-H]^-^: 359→ 223 (20), 197 (30), 179 (30), 161 (100) MS^3^ [M-H]^-^: 359→197 (100), 135 (18)	Rosmarinic acid	ME-LHv, ME-SHv
P2	29.94	322, 247, 234	537	MS^2^ [M – H]^-^:537 → 493 (100), 417 (13), 399 (60), 357 (5), 298 (10) MS^3^ [M – H]^-^: 537 →493 →359 (100), 313 (15), 295 (38), 179 (5) MS^4^ [M – H]^-^:537 →493 →359→ 223 (20), 197 (30), 179 (18), 161 (100), 135 (5)	3′-*O*-(8″-*Z*-caffeoyl)-rosmarinic acid	ME-SHv
P3	33.9	295, 241			Unknown	ME-LHs
P4	35.68	329, 295, 247			Unknown	ME-LHs ME-SHs
P5	36.2	335, 266, 243			Unknown	
P6	36.72	323,289, 245			Unknown	ME-LHs ME-SHs

a Under the evaluated conditions, MS^n^ fragmentation of P3, P4, P5 and P6 was not possible.

Based on the content of flavonoids (mg RE/g d.w.) and phenolic acids (mg CAE/g d.w.) determined by HPLC, we ordered the ME as ME-LHs (357.88) > ME-LHv (209.19) > ME-SHv (55.74) > ME-SHs (52.94) and ME-LHs (238.58) > ME-SHv (129.30) > ME-SHs (64.27) > ME-LHv (26.68), respectively.

### Biological activities

#### Antioxidant activity

The ME of *H. vegae* stems (ME-SHv) showed the highest antioxidant activity estimated by the four methods, and its values were 3–14 times higher than those of the ME with the lowest activity. The extract of *H. sinaloensis* leaves (ME-LHs) was second in antioxidant activity measured by all assays, except for the β-carotene discoloration (Table 3). The antioxidant activity of the ME-SHv, expressed as IC_50_ values, was 218 μg/mL (DPPH) and 224.9 μg/mL (ABTS).

**Table 3.** Antioxidant activity of ME from leaves (L) and stems (S) of *Helicteres vegae* (Hv) and *Heliopsis sinaloensis* (Hs)^a^.

	Evaluated by			
Methanol extract	DPPH^b^	ABTS^b^	ORAC^b^	β-carotene discoloration[1 mg/mL]^c^
*Helicteres vegae*				
Leaves (ME-LHv)	32.20 ± 2.43#	102.96 ± 8.55#	270.92 ± 19.45*	22.77 ± 0.03&
Stems (ME-SHv)	176.19 ± 12.55ξ	304.67 ± 10.25ξ	1336.6 ± 90.03&	45.02 ± 0.02ξ
*Heliopsis sinaloensis*				
Leaves (ME-LHs)	51.57 ± 2.85&	286.07 ± 11.20&	865.63 ± 13.79#	3.24 ± 0.006*
Stems (ME-SHs)	16.81 ± 0.83*	90.92 ± 6.84*	321.83 ± 32.71*	13.32 ± 1.60#
BHT				88.38 ± 2.06δ

a Results are the mean ± standard deviation of three independent replicates. Different symbols in the same column show significant differences, *p* < 0.0001 (LSD: DPPH = 7.904; ABTS = 11.273; ORAC = 59.44; β-carotene discoloration = 5.35).

b μmoles of Trolox Equivalents/g of flour on a dry weight basis (μmol TE/g d.w.).

c Percentage of antioxidant activity (% AA).

#### Antimutagenic activity

The ME evaluated up to 1000 μg/mL were neither toxic nor mutagenic against *S.typhimurium* YG1024 (0.6 ≤ MI ≤2.0) (Table 4). The number of induced and spontaneous revertants was about the same.

**Table 4.** Mutagenicity index (MI) and antimutagenicity (% of inhibition) of the methanol extracts (MEs) from Leaves (L) and Stems (S) of *Helicteres vegae* (Hv) and *Heliopsis sinaloensis* (Hs). The mutagenic agent 1-nitropyrene (1-NP) and the tester strain *Salmonella enterica* serovar Typhimurium YG1024 were used in the assay.

Methanol extract	Concentration [μg/plate]	MI^a^	Revertants/plate^a^	% of inhibition^a^^,b^
*Helicteres vegae*				
Leaves (ME-LHv)	1000	1.08 ± 0.2	135 ± 25	85.7 ± 4.67ξ
	500	1.21 ± 0.17	203 ± 43	78.5 ± 7.6#,&
Stems (ME-SHv)	1000	1.27 ± 0.22	236 ± 52	74.9 ± 9.10#
	500	0.99 ± 0.06	351 ± 9	63.3 ± 4.46*
*Heliopsis sinaloensis*				
Leaves (ME-LHs)	1000	1.14 ± 0.16	158 ± 3	83.4 ± 2.76&,ξ
	500	0.16 ± 0.3	243 ± 22	74.4 ± 6.03#
Stems (ME-SHs)	1000	1.28 ± 0.46	170 ± 4	82.1 ± 3.05&,ξ
	500	1.26 ± 0.23	227 ± 15	76.4 ± 1.94#
1-NP [100 ng/plate]			965 ± 142	

a Results are the mean ± standard deviation of two independent experiments per triplicate.

b Inhibition of the 1-NP (100 ng/tube) mutagenicity by the corresponding ME. Different symbols show significant differences (LSD = 5.67 *p* < 0.0001). The number of colonies due to spontaneous reversion was 69 ± 16.

The 1-NP treatment of *S.typhimurium* YG1024 exhibited a dose–response effect. The chosen concentration of 1-NP for the antimutagenicity evaluation was 100 ng/tube, because the number of induced revertants at this concentration was five times higher than that of spontaneous revertants.

The ME showed strong inhibition of the 1-NP mutagenicity (63–86%) at both concentrations evaluated (500 and 1000 μg/plate), and their activities differed significantly between the plants and tissues (*p* < 0.05) (Table 4). The highest antimutagenic activity was observed in the extract of *H. vegae* leaves (ME-LHv) and the lowest in the extract of *H. vegae* stems (ME-SHv).

#### Antimicrobial activity

The ME (1 mg/mL) of *H. vegae* and *H. sinaloensis* were not active against the evaluated human pathogenic bacteria and *Giardia lamblia.*

#### Correlation between biological activities and phenolics content

The total phenolics content (TP) showed high positive correlations with the antioxidant activity by DPPH (*r* = 0.8798, *p* = 0.0040), ABTS (*r* = 0.9246, *p* = 0.0010) and ORAC (*r* = 0.9965, *p* < 0.0001), but no significant correlation was found with the AA obtained by using the β-carotene discoloration method (*r* = 0.4896, *p* = 0.2182). TP also showed a positive correlation with the antimutagenic activity, but in the limit of significance (*r* = 0.7030, *p* = 0.052). However, when the correlation analysis was done with the content of the main phenolics determined by HPLC, the biological activities were neither correlated with flavonoids (0.02 ≤ *r* ≤ 0.54) nor with phenolic acids (0.01 ≤ *r* ≤ 0.68); moreover, the sum of flavonoids and phenolics acids for each extract did not correlate with the biological activities (0.16 ≤ *r* ≤ 0.58).

#### Toxicity

In the *A. salina* assay, the ME-LHv showed minimal toxicity (LC_50_ = 807.11 ± 145.6 μg/mL), and the ME-SHv was non-toxic (LC_50_ > 2000 μg/mL). In agreement with these results, we observed during field work that *Helicteres vegae* plants were consumed by cattle. On the other hand, the ME of *H. sinaloensis* showed high toxicity (ME-LHs, LC_50_ = 94.9 ± 1.7 μg/mL; ME-SHs, LC_50_ = 89.03 ± 4.42 μg/mL).

## Discussion

### Phytochemical analysis

The phytochemicals found in the studied species were similar to those found in related plants. *Heliopsis oppositifolia* has flavonoids, tannins, triterpenes/steroids and alkaloids in the aerial parts (Sanabria-Galindo et al. 1997). The fruit of *Helicteres isora* contains carbohydrates, proteins, tannins, phenolics and steroids (Tambekar et al. 2008); whereas the root has tannins, phenolics, amino acids, carbohydrates, phytosterols, triterpenoids and alkaloids (Tiwari et al. 2010). The analysis of our ME did not show the presence of alkaloids, perhaps because they are absent or below the limit of detection.

### Phenolics determined by colorimetric methods

The differences in TP found in our ME were partially associated with differences in the content of tannins; ME-SHv showed the highest content of condensed tannins (603.71 ± 1.11 mg CE/100 g d.w.) and TP (2245.82 ± 21.45 mg GAE/100 g d.w.). However, the TP content of the ME-SHv was lower than that reported for fruits of *Helicteres isora* (2600 mg TAE/100 g d.w., as tannic acid equivalents) (Loganayaki et al. 2013).

### Phenolics determined by HPLC-DAD-ESI-MS

The flavonoid F1 (*t*_R_ = 32.03 min) was only detected in *H. vegae* (ME-LHv and ME-SHv). Mass spectrometric data were: molecular ion 593 [M − H]^−^; MS^2^*m/z* 285 [(M − H) − 146 − 162]^−^ (base peak) that corresponds to kaempferol aglycone ion [Y_0_ − H]^−^; and *m/z* 447 [(M − H) − 146]^−^ identified as kaempferol 7-*O*-hexoside ion (Figure 2). A fragment of 162 U is characteristic of hexosides, whereas 146 U could be coumaroyl or rhamnosyl; coumaroyl was confirmed with the fragment *m/z* 307 [coumaroylhexoside − H]^−^. These data coincide with those previously published by Gouveia and Castilho (2010); likewise, MS^3^ of *m/z* 285 gave the fragments *m/z* 257, 255, 229, and 151 that have been reported for kaempferol (Gouveia & Castilho 2010); moreover, the identity of the kaempferol aglycone was validated with the MS data of the commercial standard. It is known that flavonols, such as kaempferol, usually present glycosyl substitutions in the 3-OH and 7-OH positions. In the MS^2^, the aglycone ion *m/z* 285 was more abundant than the aglycone radical ion *m/z* 284, and the spectra showed an intense peak for *m/z* 257 [Y_0_ − CO]^−^; these results agree with the data published by Ablajan et al. (2006), who mentioned that substitution is in 7-OH. Consequently, F1 was identified as kaempferol 7-*O*-coumaroylhexoside.

The flavonoids F2 (*t*_R_ = 27.92 min) and F3 (t_R_ = 28.47 min) of the *H. sinaloensis* leaves (ME-LHs) showed the same ion *m/z* 593 [M − H]^−^. MS^2^ showed fragments of *m/z* 447 [(M − H) − 146]^−^ and *m/z* 285 [(M − H) − 146 − 162]^−^ (Figure 1S) assigned to rhamnosyl and hexosyl units, respectively; rhamnosyl was assigned based on the absence of *m/z* 307 (Gouveia & Castilho 2010). Considering the fragmentation pattern, previous reported data (Gouveia & Castilho 2010), and the MS^3^ fragmentation of ion *m/z* 285, the results were similar to those of the commercial standard kaempferol. Therefore, F2 and F3 were identified as kaempferol 7-*O*-rhamnosylhexoside. The differences in the retention times and maxima of absorption of F2 and F3 could be associated with differences in the substituent sugars. This is the first report about the identification of phenolics in the genus *Heliopsis*.

The phenolic acid P1 (*t*_R_ = 28.93 min) showed the following MS data: molecular ion *m/z* 359 [M − H]^−^ (Figure 2S); MS^2^ produced fragments of *m/z* 223, 197, 179 and 161; and MS^3^ of m/z 197 showed an ion of *m/z* 135 that corresponded with the fragmentation of the commercial standard for caffeic acid. These data were compared with those in the literature, and P1 was identified as rosmarinic acid (Guan et al. 2014). This compound is present in *Helicteres isora* (Satake et al. 1999).

The phenolic acid P2 (*t*_R_ = 29.94 min) was found in *H. vegae* stems (ME-SHv) and showed the following MS data: molecular ion *m/z* 537 [M − H]^−^ (Figure 3S); MS^2^ produced a fragment of *m/z* 493 [(M − H) − 44]^−^ that was associated with the loss of a carboxylic group, characteristic of phenolics (Nowacka et al. 2014); MS^3^ showed the ion *m/z* 359 [(M − H] − 178]^−^, characteristic of rosmarinic acid; and MS^4^ of *m/z* 359 (i.e., 223, 197, 179, 161, and 135) validated its identity (Guan et al. 2014). Taking into account the UV spectra, the MS^n^ data and previous reports, P2 was tentatively identified as 3′-*O*-(8″*-Z*-caffeoyl) rosmarinic acid.

UV spectra of P3, P4, P5 and P6 suggested they are phenolics, but the MS analysis did not allow us to identify them (Table 2). They showed MS ions in the range of *m/z* 785–1369; however, a proper MS^n^ fragmentation was not acquired under the analyzed conditions.

### Antioxidant activity

The antioxidant activity (AA) of ME-SHv by DPPH was higher than that reported previously in ME of *Helicteres isora* fruits, whereas the opposite was observed for the AA by ABTS (Loganayaki et al. 2013). In addition, the AA of ME-SHv by the βCD method was similar to that of the aqueous extract of *Helicteres isora* fruits (Suthar et al. 2009). The high AA of the ME-SHv could be due to its content of TP and tannins. The correlation between AA and TP content has been previously reported, e.g., for flaxseed products (*r*^2^ = 0.963, *p* < 0.01) (Velioglu et al. 1998) and for three species of *Echeveria* (*r* = 1.00, *p* = 0.03) (López-Angulo et al. 2014). Our results showed a significant correlation between AA and TP determined by the Folin-Ciocalteu spectrophotometric method, but not between AA and the content of the main phenolics determined by HPLC. Thus, the AA could be due to compounds of other families, to phenolics in minor quantities in the ME, or to synergic effects.

Rosmarinic acid (P1) and its derivative (P3) have shown good AA (Cervellati et al. 2002; Zhu et al. 2014; Govindaraj & Sorimuthu Pillai 2015). The ME from the stem of *H. vegae* (ME-SHv) showed the highest content of these compounds and the highest AA. Therefore, rosmarinic acid and its derivative appear to have an important role in the AA of the ME of *Helicteres vegae*.

### Antimutagenic activity

ME of the studied plants were strong antimutagens with an inhibition of 1-NP mutagenicity in the range of 60–90% (Wall et al. 1988). Compared with the antimutagenicity values reported for plants using the same strain and mutagen, our results are very promising; the mutagenicity inhibition by ME of *H. vegae* and *H. sinaloensis* were higher than those obtained for the aqueous (500 μg/plate, 53%) and ME (1000 μg/plate, 32%) of *Randia echinocarpa* fruits (Santos-Cervantes et al. 2007; Cano-Campos et al. 2011). Moreover, these values were also higher than those reported for affinin extracted from the roots of *Heliopsis longipes* (TA98 strain, 2-aminoanthracene at 25 and 50 μg/plate) (Arriaga-Alba et al. 2013). The YG1024 strain used in the present study is derived from the TA98 strain (Watanabe et al. 1994).

The antimutagenic activity of rosmarinic acid was previously determined using *S.typhimurium* TA-100 and the mutagens sodium azide and *N*-methyl-*N′*-nitro-*N*-nitrosoguanidine (Vattem et al. 2006); the antimutagenicity of this compound was associated with modulation of redox conditions in the bacteria. In another report, rosmarinic acid reduced the risk of chromosome damage induced by doxorubicin in the micronucleus assay in mice (Andrade et al. 2008). This suggests that rosmarinic acid is partially responsible for the antimutagenicity of the ME of *H. vegae*. However, ME-LHv and ME-SHv showed similar activities but different contents of this compound; additional studies are necessary to elucidate other compounds contributing to the antimutagenicity of *H. vegae* extracts.

### Antimicrobial activity

ME (1 mg/mL) of *Helicteres vegae* and *Heliopsis sinaloensis* were not active against the evaluated human pathogens (bacteria and *G. lamblia*), because plant extracts with MIC values higher than 1 mg/mL (antibacterial assay) and IC_50_ values higher than 500 μg/mL (anti-*Giardia* assay) are considered inactive (Rios & Recio 2005; Amaral et al. 2006). Nevertheless, some researchers have reported antimicrobial activity for other species of *Helicteres* (Truiti et al. 2005; Tambekar et al. 2008; Gairola et al. 2013) and *Heliopsis* (Gutierrez-Lugo et al. 1996).

### Toxicity

The toxicity of ME from *Helicteres vegae* was slight (ME-LHv) or null (ME-SHv) in the *A. salina* model, but it was high for *H. sinaloensis* (ME-LHs and ME-SHs). Previous studies showed that the ME of *Heliopsis longipes* roots was more cytotoxic (LC_50_ = 58.65 μg/mL) than our extracts in this assay (Gutierrez-Lugo et al. 1996). *Artemia salina* assay is commonly used to establish the toxicity of plant extracts and to screen for antitumoral extracts and compounds (Meyer et al. 1982; Ajoy & Padma 2013; Tulsi et al. 2014). Thus, ME of both plants have the potential of being antitumoral agents.

## Conclusions

The ME of *Helicteres vegae* and *Heliopsis sinaloensis* showed high antioxidant and antimutagenic effects but no antimicrobial activity. The main phenolics in these plants were flavonoid derivatives of kaempferol and phenolic acid derivatives of caffeic acid (e.g., rosmarinic acid). The content of the main phenolics did not fully explain the antioxidant and antimutagenic activities, but the contribution of the rosmarinic acid derivatives could play an important role. In general, *H. vegae* extracts were non-toxic, whereas *H. sinaloensis* extracts showed selective toxicity: high toxicity was shown in the *Artemia salina* assay, and they were nontoxic in the Ames assay and against the evaluated microorganisms. Our results suggest that *H. vegae* and *H. sinaloensis* could be a source of novel therapeutics or supplements for the treatment and prevention of chronic-degenerative diseases.

## Supplementary Material

Francisco_Delgado-Vargas_et_al_supplemental_content.docx
