# Supplementary material for: Chemical composition and biological activities of Helicteres vegae and Heliopsis sinaloensis
Source: Pharm Biol. 2017 Mar 28;55(1):1473–82. doi: 10.1080/13880209.2017.1306712 (PMC6130667; doi:10.1080/13880209.2017.1306712)
Supplement: Francisco_Delgado-Vargas_et_al_supplemental_content.docx [file IPHB_A_1306712_SM4691.docx]

**Figure 1S**

| 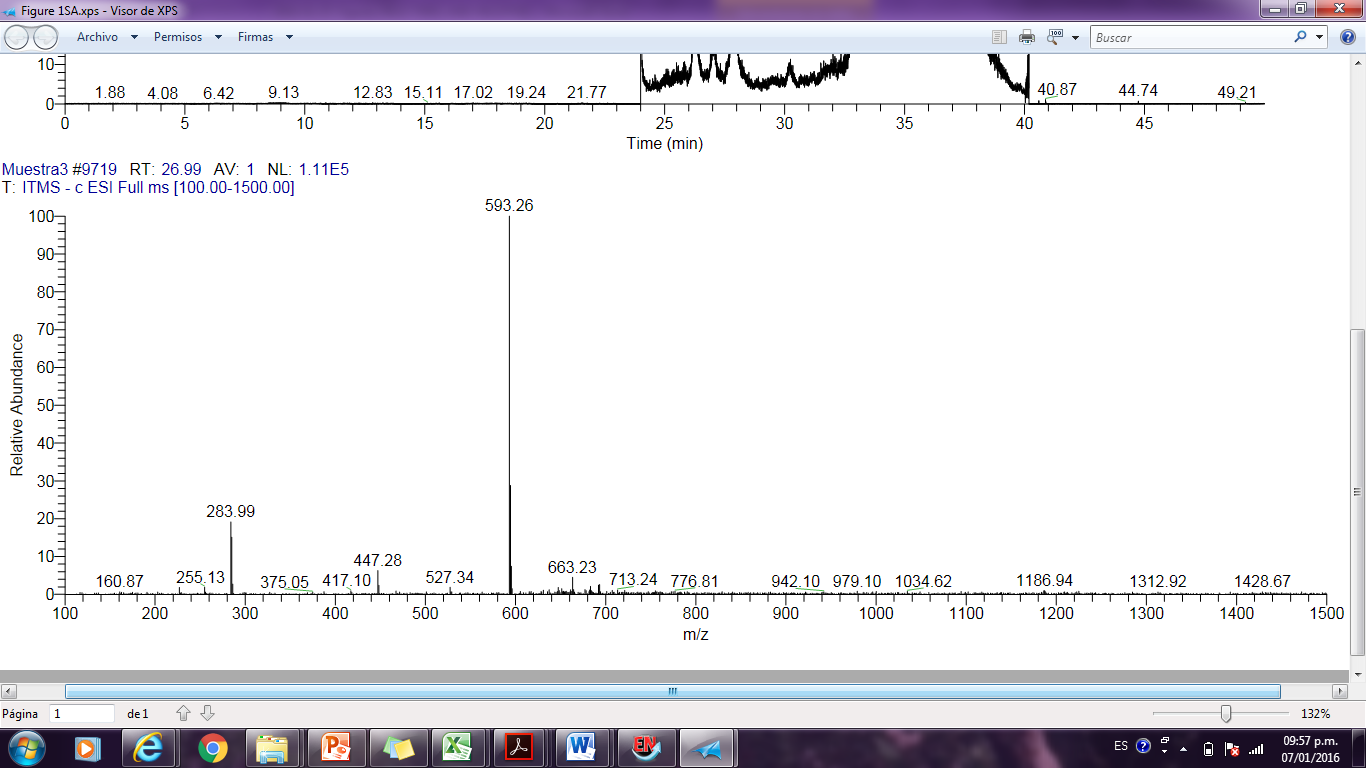  **(A)** |
| --- |
| 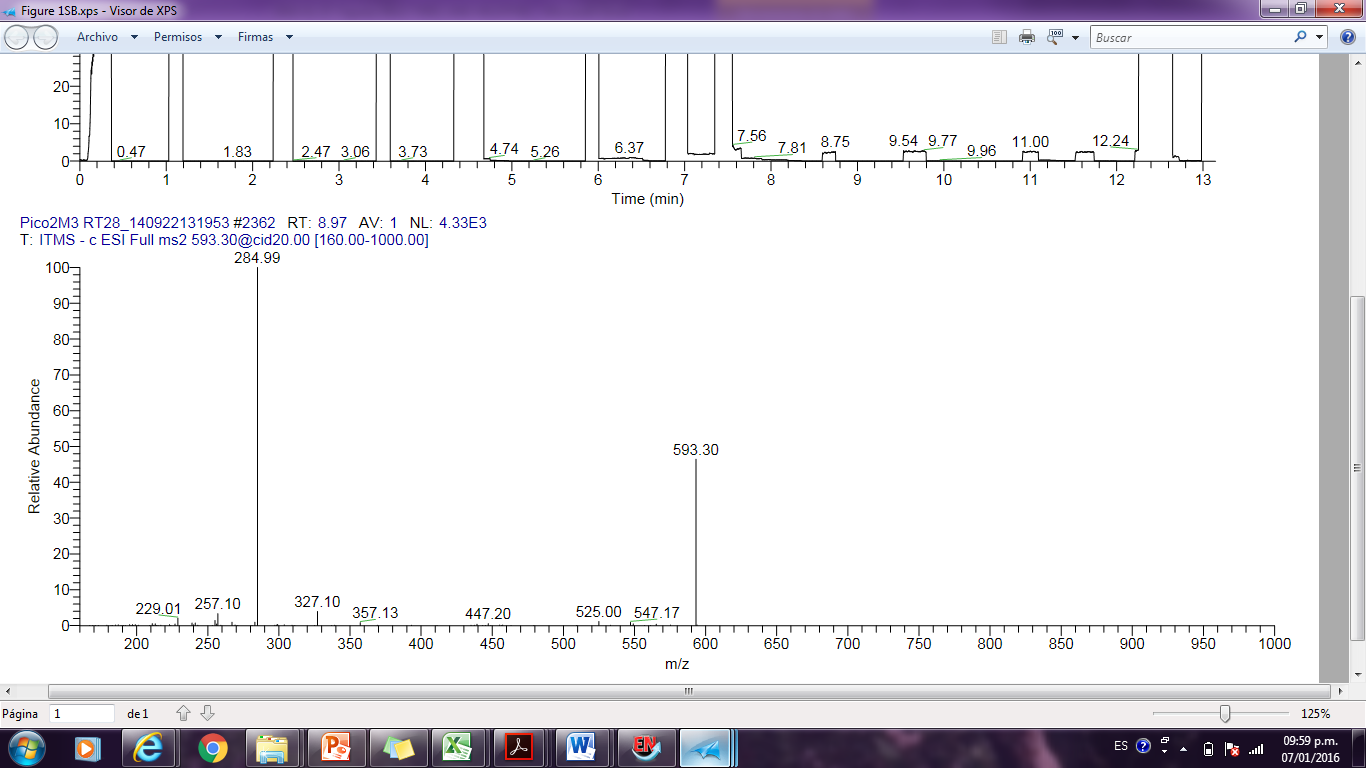  **(B)** |
| 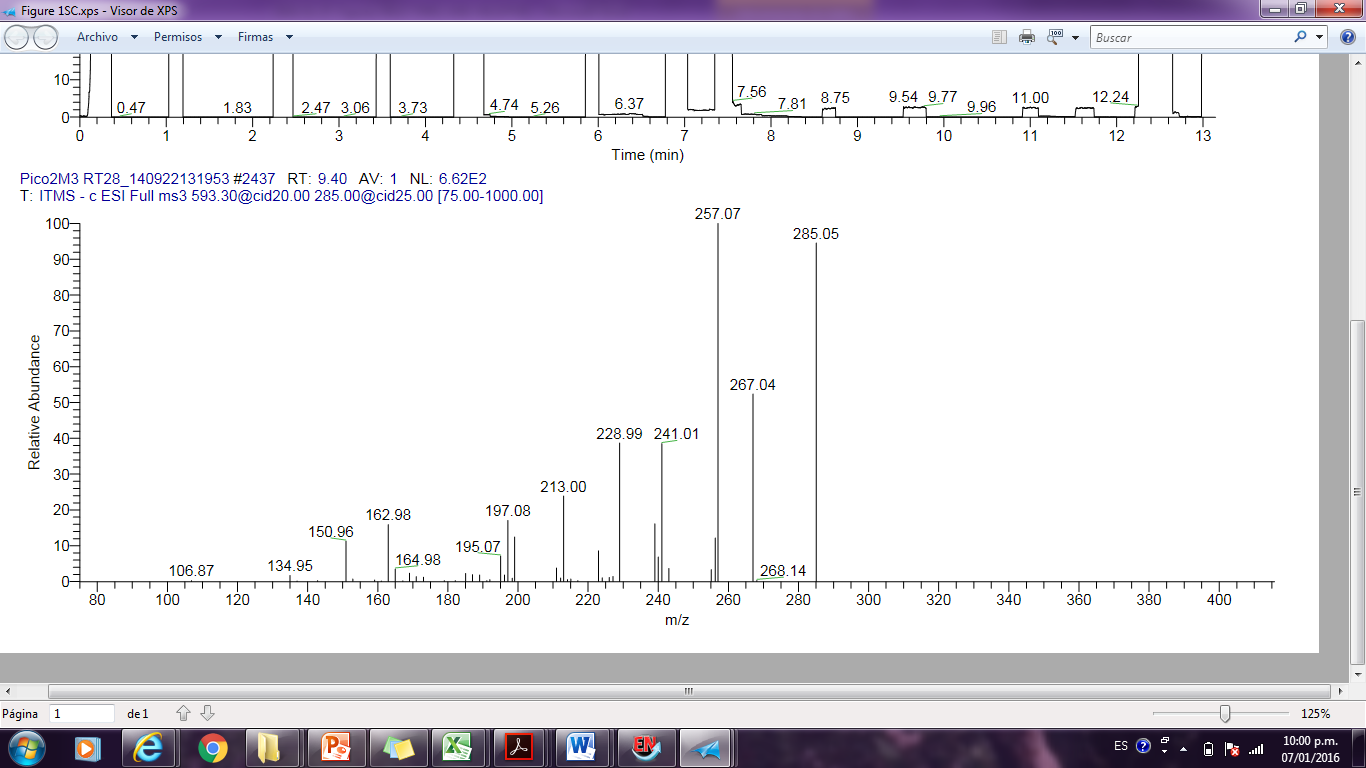  **(C)** |
|  |

**Figure 1S.** ESI-MSn in negative mode of compound F2. Full scan (A) and

fragmentation of ion m/z 593, MS2 (B) and and MS3 (C).

| **Figure 2S** |
| --- |
| 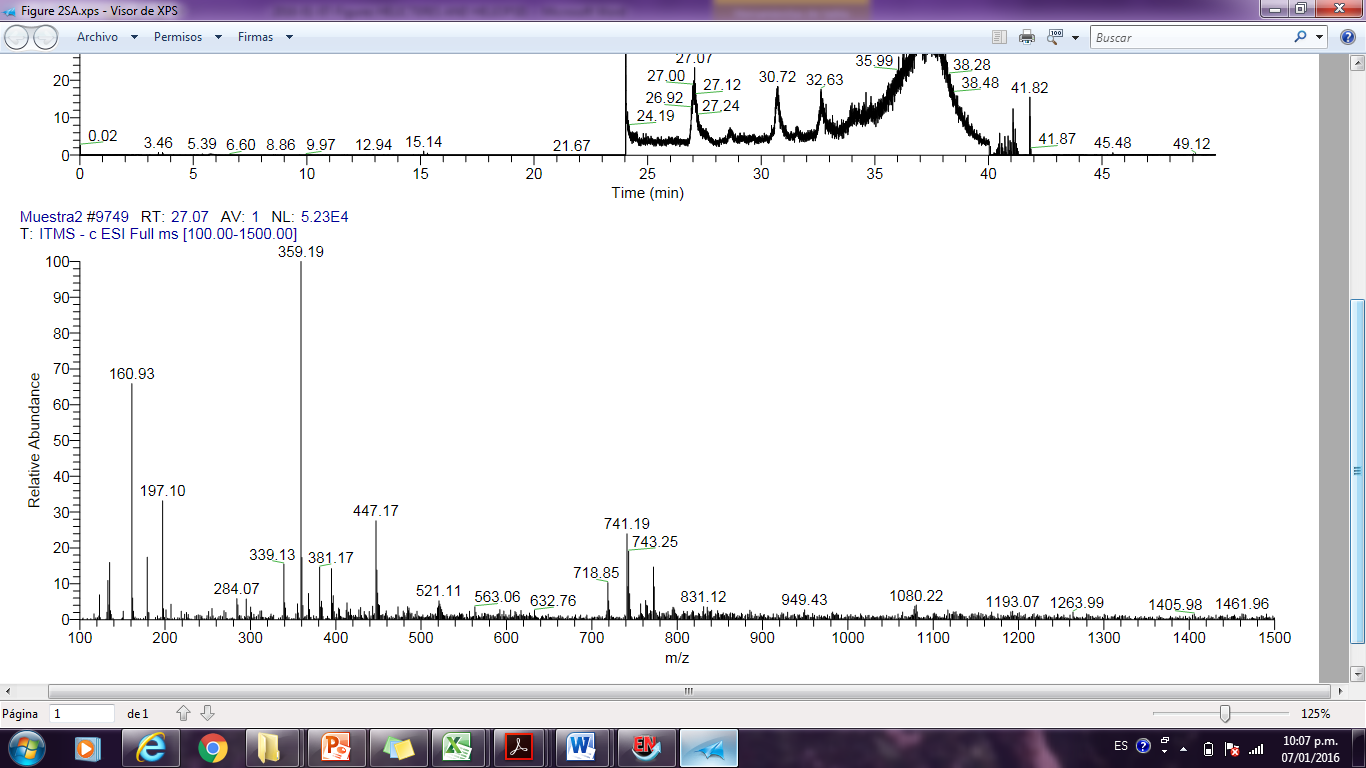  **(A)** |
| 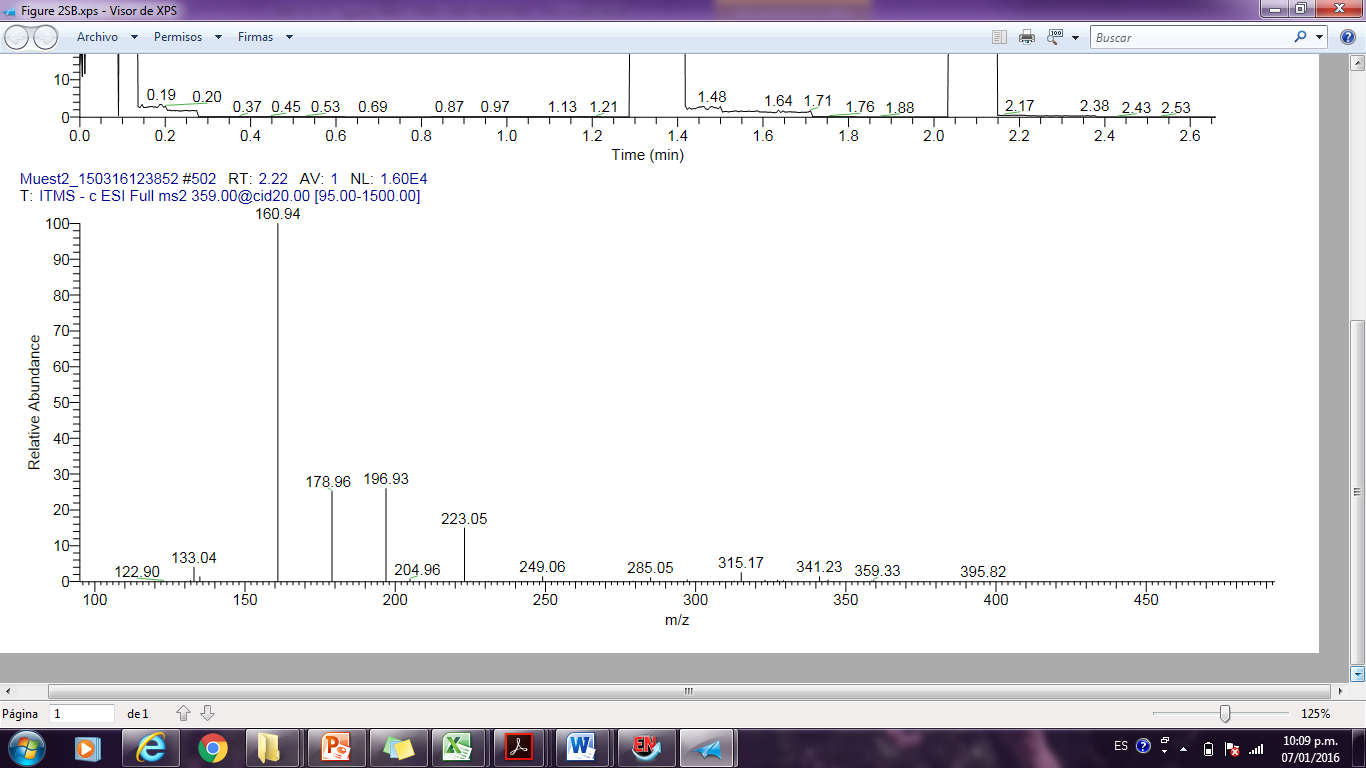  **(B)** |
| 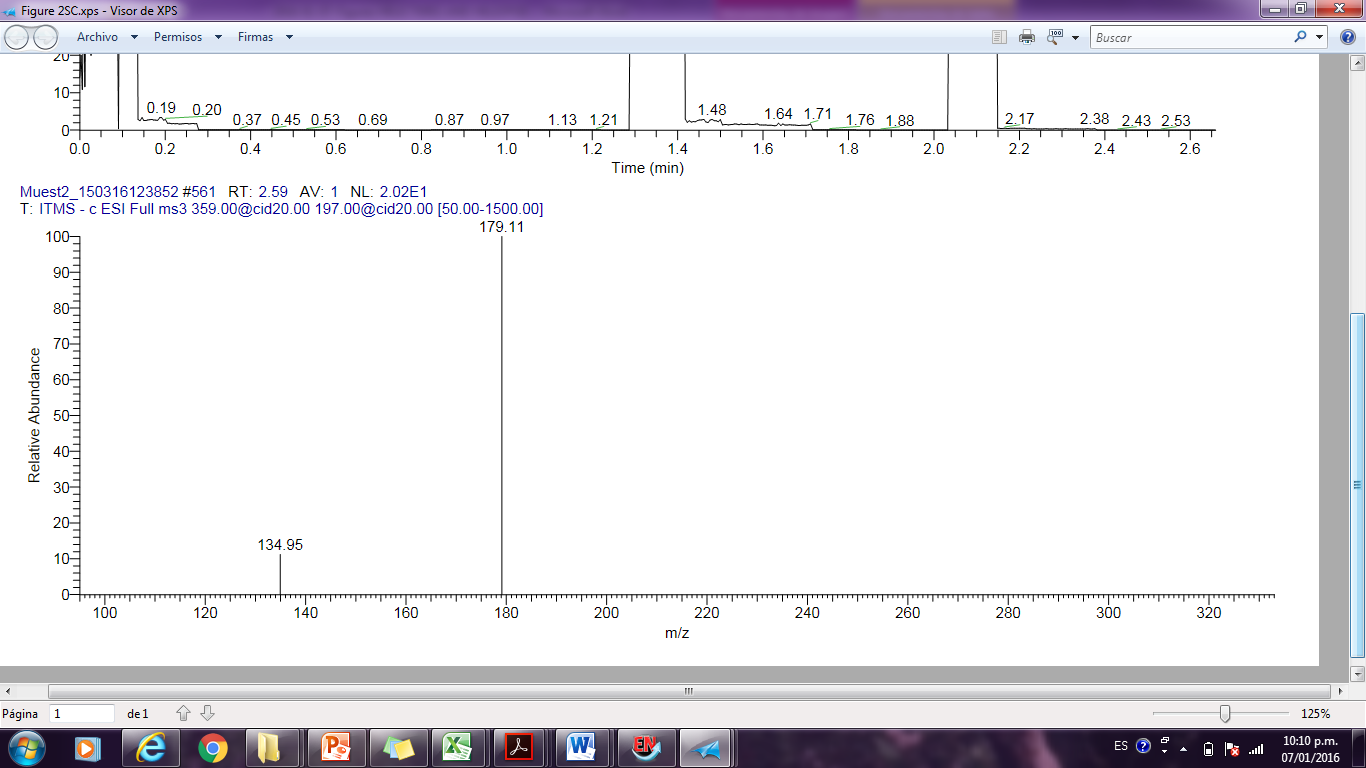  **(C)** |
|  |

**Figure 2S.** ESI-MSn in negative mode of compound P1. Full scan (A) and

fragmentation of ion m/z 359, MS2 (B) and MS3 (C).

**Figure 3S**

**
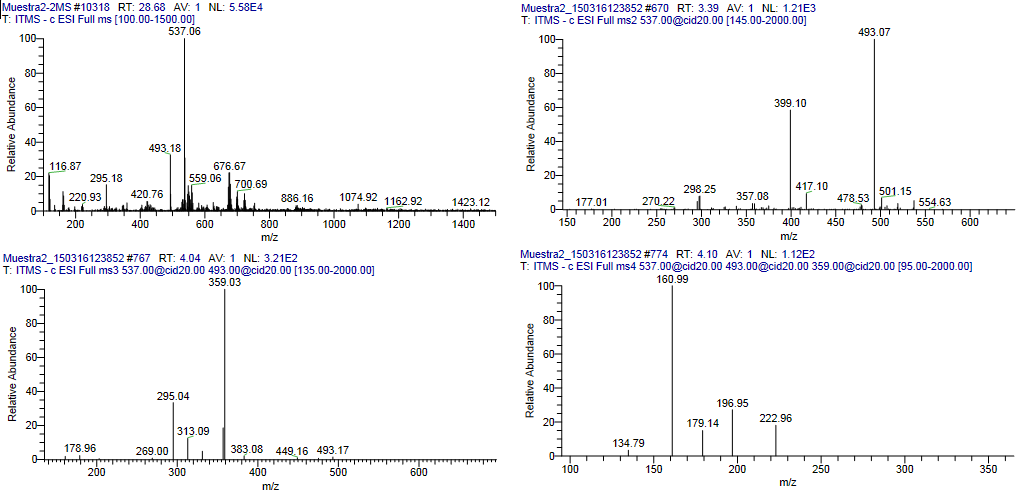
**

**(A)**

**(D)**

**(B)**

**(C)**

**Figure 3S.** ESI-MSn in negative mode of compound P2. Full scan (A) and fragmentation of ion m/z 537, MS2 (B), MS3 (C), and MS4 (D).
